# ‘This year I will not put her to work’: the production/reproduction nexus in Burkina Faso

**DOI:** 10.1080/13648470.2012.692360

**Published:** 2012-08-17

**Authors:** Katerini Tagmatarchi Storeng, Mélanie Stephanie Akoum, Susan F. Murray

**Affiliations:** a Centre for Development and the Environment/Institute of Health and Society, University of Oslo, Oslo, Norway and London School of Hygiene & Tropical Medicine, London, UK; b Africsanté, Bobo-Dioulasso, Burkina Faso; c King's College London, London, UK

**Keywords:** medical anthropology, reproductive health, Burkina Faso, illness meaning, women's work

## Abstract

Global advocacy campaigns increasingly highlight the negative impact of reproductive morbidity on economic productivity and development in order to justify donor investment in maternal health. Anthropological approaches nuance such narrow economic estimations of reproductive health. Drawing on ethnographic fieldwork from Burkina Faso in West Africa, this paper analyses the dynamic, and sometimes contradictory, relationship between women's work and reproductive health in impoverished communities. Specifically, it examines the consequences of life-threatening ‘near-miss’ obstetric complications for women's work across domestic, agricultural and economic spheres over a four-year period. Such events provide a window onto the diverse ways in which production and reproduction are intimately linked within women's everyday lives. Reproduction and production entail sources of potential empowerment and enhancement, as well as potential threats, to health and well-being. In the aftermath of ‘near-miss’ events, the realms of reproduction and production sometimes jeopardise each other and at other times reinforce each other, while strength in one domain can compensate for weakness in the other. Women's experiences thus reveal how ‘production’ and ‘reproduction’ are mutually constituted, challenging the purely instrumental accounts of pregnancy-related ‘productivity loss’ that dominate current global health discourse.

## Introduction

Global advocacy messages urge donors to ‘invest’ in women's health because ‘it pays.’ Such calls are justified with reference to global estimates that $15 billion is lost every year due to reduced productivity related to the death of mothers (Gill, Pande, and Malhotra 2007). Advocacy specialists also point to evidence that pregnancy-related ill health can drain family resources and savings, change patterns of consumption, reduce households to debt and poverty, and further impoverish those who are already poor (Xu et al. 2003). Investment in women's health, it is argued, therefore makes economic sense because pregnancy-related ill health reduces the time women spend in paid work, in turn diminishing household resources and exacerbating economic insecurity (Hutton 2006). This recent attention to the interface between reproductive morbidity and economics aims to situate maternal health very explicitly within poverty reduction justifications for health investment that have emerged as an important element of the global health discourse as part of the Millennium Development Goal (MDG) framework (Standing 2002).

Against such instrumentalist economic framings, anthropological approaches can help to position women's health in a broader context and demonstrate the nonlinear, dynamic and sometimes contradictory relationship between reproductive health and work. Anthropological research shows that ‘work’ is rarely confined to formal-sector employment (Manderson 1983). Meanwhile, health problems often ‘travel’ through the different domains of life and work (domestic, informal and formal work), while notions of well-being are constituted through multiple social networks that cut across places of work (Prentice and Day 2010). Consequently, anthropologists have recently called for a critical reconceptualisation of the everyday politics of labour within the contemporary context of trade liberalisation, the deregulation of financial markets and the informalisation of labour (Prentice and Day 2010).

Such calls recognise previous ethnographic analyses of afflictions ranging from repetitive strain injury to spirit possession as forms of embodied resistance to punishing labour conditions (Prentice 2009; Lock 1990; Scott 1985). Ethnographies of women's health, in turn, draw attention to the prominent position of work within individual explanatory frameworks of ‘reproductive disruptions’ (Inhorn 2007). Chapman's (2003) research from Mozambique, for instance, shows that women see their own reproductive morbidity as being intensified by poverty and the burden placed on them for family subsistence and continuous fertility. Similarly, Ghanaian women understand their reproductive health as being shaped by their heavy work loads, resulting from gender relations that constrain them from achieving a measure of economic independence and predictability (Avotri and Walters 1999).

Scholars have also started to place more positive emphasis on social resilience in the face of challenging livelihood conditions, including those resulting from acute health crises within heterogeneous and rapidly changing settings, in which structural and environmental forces fail to create security for sustaining life in the event of crisis (Obrist, Pfeiffer, and Henley 2010; Obrist 2003). Drawing on Bourdieu's (1977) practice theory, Obrist and colleagues draw attention to the material and non-material resources – economic, social and cultural capital – that structure human capacity to act in view of threats such as illness. Taking into account Bourdieu's concept of social fields – the configuration of social positions held by individuals – further helps to capture the notion that actors have different configurations of ‘capitals’ and are therefore differently affected by similar hazards and face varied constraints and opportunities in building resilience.

Building on this literature, this article makes the link between reproduction and production explicit by using women's experience of severe and life-threatening ‘reproductive mishaps’ – to use Bledsoe's (2002) term – as a window onto the diverse ways in which production and reproduction are intimately linked within women's everyday lives. Drawing on longitudinal ethnographic research from Burkina Faso, the paper analyses the impact on women's work of ‘near-miss’ obstetric complications in impoverished, peri-urban communities. Obstetric ‘near-misses’ are complications relating to pregnancy, abortion or delivery that are so severe that women likely would have died in the absence of urgent medical intervention (Mantel et al. 1998). Women's experiences of ‘near-miss’ events reveal how ‘production’ and ‘reproduction’ are mutually constituted in Burkina Faso, and highlight how the gendered power dynamics of women's work sometimes place women in a double bind. The impact of severe reproductive morbidity thus goes well beyond the economic ‘productivity loss’ that is the focus of current global health advocacy.

## Background

**Figure 1.**
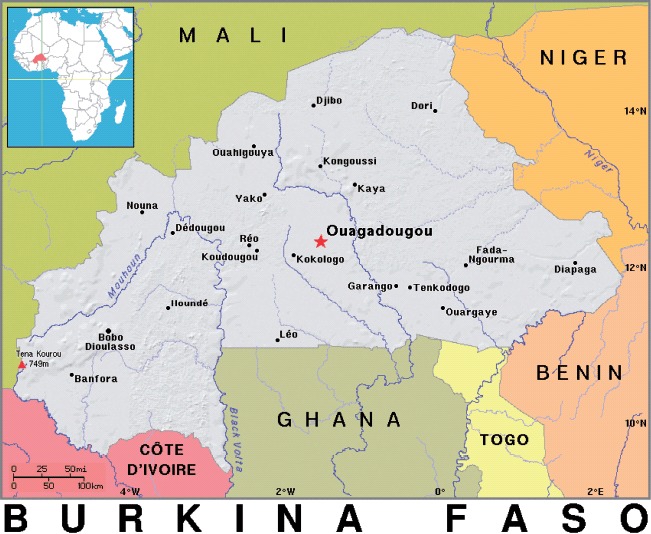
Burkina Faso's geographical location.

Burkina Faso, landlocked in West Africa (Figure 1), is one of the world's poorest countries (UNDP 2009). The Mossi are the dominant group in an ethnically diverse country. Traditionally, social organisation has been patrilineal, dominated by descent-based farming units, but today household arrangements range from single-person to multi-generational and extended households (Helmfrid 2004). Polygyny is frequent in both rural and urban areas, including formal polygamy, co-habitation in extended households and informal forms of polygamy in which men establish several geographically separate nuclear households (cf. Chapman 2003). While half of the population is classified as Muslim, gender segregation is much less strict than in Arab countries (Helmfrid 2004), as reflected in the high female labour participation rate, which was 82% in 2008 (International Labour Organisation 2009).

Patterns of women's work reflect those described elsewhere in West-Africa, where women have traditionally been appointed custodians of family welfare and health, and are socialised from a young age into cleaning, sweeping, nursing and caring for the younger children, the aged and the sick, often at the expense of formal education and training (Kabira, Gachukia, and Matiangi 1997). Women have also historically played an important role in agriculture, but the growth of cash crop production and trade after colonisation of West Africa gradually marginalised them in the subsistence sector, with the result that their productivity and control over resources decreased at the same time that their total work burden increased (Lado 1992). Today, people in Burkina Faso survive primarily on maize, millet or other grains grown in household fields, and most Burkinabé women contribute to some agricultural production.

Since the 1980s, structural adjustment reforms and trade liberalisation have created new opportunities for formal employment, although these are mostly insecure, poor-quality jobs. Most households now combine subsistence agriculture with small-scale trade or seasonal wage-labour, notably for the state-owned cotton company (see Ouédraogo and Fofana 2009). As in other parts of Africa (cf. Loewenson 2001), women have had poor access to ‘modern’ sector employment, and are often confined to the informal economy and activities such as street vending or trading, where they lack protection against injury, illness or market failures. With increased poverty, they have also taken on heavier workloads as males migrate to urban areas and neighbouring countries in search of work.

The vulnerability associated with women's uncertain economic futures is intensified by their poor reproductive health. Burkinabé women have an estimated one in 28 lifetime risk of maternal death (WHO 2010). This reflects social and economic disadvantage, but also that the poorly functioning district health system does not adequately address availability of comprehensive obstetric care (Hounton et al. 2005). Moreover, such care is often unaffordable (Storeng et al. 2008), although the recent introduction of a government subsidy for facility-based delivery may be improving access (De Allegri et al. 2011).

## Methods

Ethnographic fieldwork was nested within a longitudinal epidemiological cohort study into the consequences of obstetric emergencies. In late 2004 and early 2005, 1014 women (337 with obstetric near-misses and 677 controls with uncomplicated deliveries) were recruited before discharge from six university, district and regional hospitals across Burkina Faso (Filippi et al. 2007). In parallel, 82 women from this cohort were then selected purposively for in-depth follow-up, the majority of whom (66) had experienced an obstetric near-miss (see Storeng et al. 2008). These complications included haemorrhage, eclampsia (pregnancy-induced hypertension and convulsions), anaemia, infections and unsafe abortion, the major medical causes of maternal mortality. In about half of these cases, the baby was stillborn or died soon afterward. The sub-sample included unmarried women, women in monogamous marriages and in polygamous marriages, from different religious and ethnic groups. They ranged in age from 14 to 45, had from none to nine previous pregnancies, and, on average, five years of schooling.

Two of the authors (KTS and MA) and another anthropologist conducted two to three in-depth interviews with each woman during the year following hospital discharge, during home visits usually lasting between one and three hours. Such visits provided opportunities for informal conversations and observations of women's daily activities and social interactions. Those women residing in the city where the study team was based were visited more regularly. Whenever possible, male partners or relatives were also interviewed. In addition, 16 of the near-miss women from two of the study sites in the western part of the country were re-interviewed by MA about four years later (2009 and 2010). Interviews were conducted in informants’ preferred language (usually Dioula, but also Mòoré and French), and were recorded and transcribed, with simultaneous translation to French. All names in the text are pseudonyms.

## Findings

### Conceptualisation of the interface of work and reproductive health

The conceptual link between pregnancy and work – broadly conceived – featured prominently in women's accounts, which variably described work as beneficial or as a potential threat to well-being. On the one hand, women explained, maintaining regular activities during the early stages of pregnancy is essential to ensuring a healthy pregnancy and that the unborn child ‘sits well,’ while activities such as street trading keep women active and well-nourished. As one woman put it, ‘if you sit all the time, the blood will accumulate in your stomach and the day of your delivery it will wear you out,’ referring to ethnophysiological ideas about the importance of balancing blood, strength and vitality during pregnancy.

As pregnancy progresses, however, women must carefully calibrate physical activity to protect the pregnancy and abandon physically demanding tasks such as chopping wood and carrying water, a view that was reinforced by medical advice to abstain from such heavy work. According to first-time mother Sanata, those who do not comply with such advice risk reprimands from healthcare providers: ‘if the child dies, they will tell you that you killed your child and that there is even a risk that you might lose your own life, because you weren't supposed to work and you still did.’

As Sanata's comment implies, within the context of intense poverty, negotiating rest from work is not always possible, and women often continue gruelling work routines throughout pregnancy. In rural areas, this might involve 18-hour days of gathering firewood from the bush, milling grain for flour, cooking, contributing to agricultural work and looking after children and the elderly, while in more urban settings long days of street vending compound heavy domestic burdens.

The economic necessity of women's unpaid labour, including agricultural work, exacerbates power differentials between men and women that can make it near impossible for women to negotiate necessary rest, as 20 year-old Fatoumata's account of her late-term miscarriage illustrates:

The pregnancy happened to coincide with harvest time when we walk and work a lot. During that period, when you tell the men that you are not feeling well, they don't see anything but their work. It's as if they don't know what illness is. That's why I miscarried… I worked too much. I suffered from a series of minor illnesses, but I was never able to rest. When I said that I was sick they thought that I was taking advantage of my pregnancy. To them I'm lazy. It was only when I had to lie down because I was not able to do anything that they started to take me seriously.

Like Fatoumata, many others attributed pregnancy-related difficulties to their heavy domestic workloads. They described adverse pregnancy outcomes as a kind of ‘occupational hazard’ of demanding daily regimes, internalising understandings of pregnancy risk within notions of work risk and reward.

Women often spoke about the ‘the heavy work of childbearing’ (’*den wolo ye barayé*,’ in Dioula), further highlighting the contingency of production and reproduction within popular aetiological frameworks. Both heavy work and childbearing, women explained, gradually wear out the body in a natural and inevitable process of depletion, which affects productivity too. As Salimata put it, When you deliver, it obviously shortens your life, because each delivery weakens you more and more. Everything is up to God. He is the one who will give you back the force to start working again.’ Traumatic childbirth takes a particularly heavy toll on the body; the combination of demanding physical activity and difficult pregnancies makes women ‘old before their time’ (’*kôrô djona*‘). These findings resonate with ethnographic evidence from the Gambia, where women see their reproductive mishaps as contingent on the cumulative physical, social and spiritual hardships of personal history (Bledsoe 2002).

## Domestic disruptions

In women's aetiological frameworks, protecting reproductive and productive capacity after a reproductive mishap depends crucially on rest from both reproductive and productive labour. However, the period of complete rest after childbirth, said to be traditionally 40 days, is often shortened considerably because of practical or economic needs. While women with uncomplicated deliveries often strap their newborns to their backs and resume their usual activities within a matter of days, those who experienced severe complications often struggle to return to household chores within an ‘acceptable’ period. While they might manage childcare, cooking and simple cleaning, more demanding tasks often proved an enduring challenge, sometimes exacerbated by inadequate access to medicines. Yacouba, for instance, could only afford to buy his wife medicines for pregnancy-induced hypertension intermittently, such that she spent regular periods incapacitated by dizziness and nausea.

Recovery after traumatic childbirth also depended on careful calibration of women's activities. This helps to explain why Yacouba was willing to forfeit Kalizeta's agricultural contribution to secure her recovery. ‘This year I will not put her to work,’ he explained. ‘I will wait until they tell me that she has recovered completely. If I allow her to work [in the fields] this year, her illness will start again.’ Kalizeta, for her part, agreed that she needed to rest, but for her the concern was to not only to secure her productive, but also her *reproductive* capacity. As she put it, ‘I need to rest – otherwise another pregnancy will never work.’

Supportive social structures are necessary for women's rest. In Kalizeta's case, her husband and co-wife willingly substituted for her in the fields and at home respectively, enabling the household to maintain a level of productive continuity despite her illness, at least in the short term. Yacouba insisted that having two wives has protective benefits, not least to maintain a gendered balance of work:

We cultivate in the bush. If a wife says that she has a headache or that she has a stomach ache, I would have to abandon my work in the fields and come back to help her find food. But if there are two of them, if one of them is absent or sick, the other one can cook for her… If there is only one wife and she falls ill, there's a big problem. The man can no longer do his own work. He will even have to take over the cooking.

Like her husband, Kalizeta – and indeed several others living in polygynous households – recognised such cooperative benefits. However, cooperation was not the norm. Many women carried out physically demanding activities in the months after surviving a ‘near-miss’ despite continued weakness and illness. For some, this was a pragmatic necessity given a lack of labour substitution. Mariame put it frankly: ‘If you don't have anyone to do it for you, what are you supposed to do? You resign yourself to God and you fetch your own water and chop your own wood.’ In other cases, however, women resumed work despite illness, to attenuate the negative social impact of the health crisis and to secure their social status within a highly competitive social structure. Indeed, the displacement of household responsibilities frequently engendered or exacerbated social tensions, especially among co-habiting women. Those unable to assume their turn in the household rota risked accusations of laziness and blame. One woman even described how her co-wife refused to cook when she herself was unable to.

Recovery of enough physical capacity to contribute to agricultural and domestic work is thus crucial to protecting women's social standing. There is a form of trade-off between personal health and work-related obligations: the one needing to be strategically negotiated in order to secure the other. Conversely, inability to contribute at the household level sometimes entails social repercussions that can themselves have impoverishing effects, for instance by reducing access to husbands’ cash and other resources.

Such tensions resulting from illness-induced domestic disruptions can be compounded by the loss of fertility, a frequent consequence of severe obstetric complications. Indeed, women were adamant that losing the ability to produce and reproduce are equally calamitous. As with infertility, ‘losing the strength of one's arms’ (’*ni muso fanga baana*‘) entails not only abuse from co-habiting women, but also the risk of male abandonment or the arrival of a second wife to compensate for the diminished household workforce (Storeng et al. 2010). Awa was only half-joking when she said that any man would take at least three wives, if he could afford it, ‘for the sake of the farm work.’ Like those who are infertile, women unable to work not only risk repudiation within the household, but may also struggle to find a new partner. As Djéneba bluntly put it: ‘if you can't work, who is going to accept you.’ For those whose pregnancies ‘fail’ to produce a living child, intensified domestic work can help protect their social position.

## The perils of productivity loss

The interface between obstetric complications and work played out differently in the *economic* sphere, where disruptions to women's income-generating work threatened not only economic well-being, but also women's sense of independence. Although most women in peri-urban areas are dependent on a daily *‘popote’* or allowance from male relatives to meet daily needs, like a large proportion of Burkinabé women, almost all the women in the study also had some form of regular income-generating activity before their hospitalisation. A minority had formal employment, such as secretarial work, but informal-sector street vending was the main occupation. Trade items ranged from vegetables, firewood, prepared foods and garments, to crafted goods such as shea butter soap. Some also farmed small plots of land independently, for subsistence and resale. A near-miss event caused long-lasting disruptions to such income-generating work through a variety of mechanisms.

A first impediment was prolonged hospitalisation and continued illness and weakness after obstetric trauma, often combined with distress following stillbirth or the death of a newborn. Several months after surviving a life-threatening puerperal infection after an incomplete miscarriage, 40 year-old Djéneba was not able to trade because, as she put it, ‘my whole body has become soft, my heart hurts and I can't work. I just lie down. Even preparing *to* [the staple maize dish] is a problem for me.’

Second, ‘catastrophic’ healthcare costs indirectly delayed women's resumption of vending and other income-generating activities, not least because a frequent coping strategy for meeting healthcare costs was sale of women's trading stock (Storeng et al. 2008). Within a context of intense economic hardship and very limited access to credit, the challenge of replenishing such stock can reinforce health-related constraints. As Natou explained six months after her near-miss: ‘I don't trade. I used to sell little things like peanuts, but since my delivery, I can't take the burden. I will wait a while before I start my activities … the problem is that I don't have the cash to get started.’ Other traders lost niche market positions to competitors following prolonged hospitalisation, while more formal income opportunities were also affected. Thirty-year-old Sylvie, for example, lost her waitressing job after lengthy hospitalisation after an unsafe abortion.

Gendered power struggles relating to women's independent work can compound such physical and financial impediments. Men often oppose women's independent activity because it threatens their authority. According to Elise, ‘men don't accept that their women move around [to trade]. It's as if when you go somewhere you are chasing after [other men], if you go out they don't like it.’ Reflecting such attitudes – to which several male informants admitted – several husbands prevented their wives from resuming their economic activities. Sometimes, this reflected genuine health concerns. However, there were also cases where men used the near-miss as a false pretence to exercise a form of ‘economic violence’ (Fawole 2008), limiting women's access to funds and credit, controlling their access to employment and excluding them from financial decision-making. Nearly a year after Salimata's near-miss, her husband refused to act as a guarantor for a loan she needed to continue her trade. Samira's husband disconnected the fridge she used in her beverage trade as a means to limit her independence during an ongoing marital dispute. Such situations create a double bind for women who feel that male contribution to the household budget is chronically insufficient. As Elise put it, ‘he won't buy me the things I need and if I manage on my own and make my own money, he gives me a hard time. It's hard work.’

The social consequences of disruptions to women's remunerated work are often as significant as the economic, particularly because sudden income reductions often increase women's dependency on men and exacerbate tensions between co-habiting women. Women valued their income-generating work highly despite it being precarious and poorly paid (daily incomes rarely exceeded 1000 to 2000 F CFA, or about US$1.90 to $3.80). Without independent income, they were no longer able to compensate for shortfalls in men's cash contributions or make discretionary expenditures on clothing, ceremonies and gifts. Instead, they had to engage in the fraught process of soliciting male partners for funds that are invariably in short supply. In addition, many said they missed trading, which, although often demanding, provides a reason to leave the domestic compound and extend social networks, imbuing daily life with a sense of interest and, frequently, fulfilment.

## Changes to the production/reproduction nexus over time

Achieving a desired balance between production and reproduction in the aftermath of near-miss events depends on the clinical nature and prognosis of the complication, but also on the social and financial capital women are able to mobilise to resume their activities in a timely manner. Social and economic support from a benefactor, whether a family member, friend, acquaintance or new partner, often facilitates access to healthcare and contraceptives, as well as access to new opportunities, cash or stock for street trading on credit, or a piece of land to farm. With such support, a number of women in the study demonstrated considerable entrepreneurial flair, starting with a small cash or stock input and gradually managing to diversify their stock and increase their income.

Continued illness and weakness can severely impede women's resumption of work. Yet those whose recovery was elusive often adapted to their changed capacities, for instance by negotiating task-sharing or additional domestic support from relatives or children. Some even eventually resumed income-generating work, although usually out of economic necessity rather than volition. They overcame physical impediments by reducing the volume of work, or shifting to other activities or forms of trade. Douba, for instance, had not regained her strength even four years after her near-miss, and, in response, had abandoned strenuous market trading in favour of the less profitable sale of home-grown vegetables from a stall outside her house. Bintou, a 30-year-old mother of five who survived eclampsia, remained unable to complete routine household chores and agricultural work four years later, but had retained some independent income by halving the volume of porridge she made to sell. Women's success in resuming income-generating work should thus not obscure that they sometimes work at lesser intensity, earn lower income and are less satisfied with their work than before the illness-induced disruption.

Pregnancy outcomes and future fertility desires also affect women's approach to productivity after near-miss events. Although many of those who recovered more slowly expressed a desire to delay pregnancy to ensure recovery, they were not always able to do so. After losing her baby during delivery, one young, recently-married woman intensified her street trading, spending almost everything she earned on fertility drugs in a bid to prove’ her fertility and to establish her position within her new household. Others, by contrast, were unable to access contraceptives, resulting in unwanted pregnancies that could have severe, and sometimes fatal, consequences (Storeng et al. 2012).

However, near-miss events can also engender a complete reversal in women's approach to the production/reproduction nexus (Murray, Storeng, and Akoum n.d.). For Djéneba, a 40-year old with two daughters, having a son had been a priority because she could not rely on support from her philandering alcoholic husband. After a life-threatening puerperal infection resulting from her fifth miscarriage, Djéneba defied medical advice to rest for two years and invested her small income on fertility treatment, only to suffer another miscarriage. Three years later, she had eventually changed her life-strategy: she had abandoned her attempts to get pregnant in order to focus on expanding her small-scale vegetable trade. Eventually, she was able to turn to more lucrative cattle rearing, and invested her income in her daughters’ education, partly to secure her own old age. Despite the primacy of both reproduction and production in women's daily lives, Djéneba's experience thus highlights that strength in one domain can, in some circumstances, compensate for weakness in the other.

## Conclusion

The interface between the consequences of obstetric complications and women's work in Burkina Faso confirms ethnographic evidence from elsewhere in Africa of women's preoccupation with protecting and maintaining their reproductive capacity (Inhorn 2007; Jenkins and Inhorn 2003; Bledsoe 2002), as well as of the importance of women's work to household welfare and individual well-being (Ouédraogo and Fofana 2009; Pike and Patil 2006; Obrist 2003). This article makes the link between reproduction and production explicit and central to the analysis of the impact of severe obstetric illness on women's work across multiple domains.

In women's narratives, reproduction and production appear as contingent processes requiring careful calibration. Pregnancy itself is internalised within notions of work risk and reward. On one level, women's work is extremely gruelling and contributes to unsuccessful pregnancies, but the inability to return to work after such complications is also problematic. Interpreting this apparent contradiction requires recognising the social meaning of work, particularly the distinction between income-generating work, which is satisfying because of its social dimensions and the economic independence it offers women, and extremely demanding domestic and agricultural work over which they have little control.

Women's experiences highlight that the trauma of pregnancy complications relates not only to the fact that such events threaten life and health, but also to the way they potentially devalue women in their two principal roles: production and reproduction. As Fassin (2001) discusses with reference to Ecuador, a major reason that many fear obstetric complications is because of a widespread belief that women who have undergone interventions for such complications, including caesarean section, are no longer capable of procreation or work, and risk repudiation. However, these connections between reproduction and production are not simply linear and causal, or a question of seeking the most effective balance between health and work. Women's activity is best understood as a series of ways of negotiating the precariousness of that balance.

The contrast between the impact of reproductive mishaps in the domestic and economic work sphere is striking. While men expect and depend on women's contribution to subsistence agriculture, food production and child care, they are less supportive of women's economic activity, sometimes even exercising ‘economic violence’ (Fawole 2008). Despite this, women go to great lengths to resume independent remunerated work after illness-induced interruptions. They do not simply negotiate health and work in order to keep healthy and to keep working, but also because their own social independence rests upon the ability to remain both well – and so potentially available for reproduction – and industrious – and so able to provide an income for themselves in the absence of strong support networks.

The varied ways that women approach the production/reproduction nexus over time reveal a variety of bargaining and strategising tactics through which they try to maintain both their productive and reproductive potential. Through these tactics, some women manifest considerable resilience, if we understand this as proactive capacities to anticipate, change and search for new opportunities’ (Obrist, Pfeiffer, and Henley 2010, 290). At the same time, it is important to avoid confusing such resilience’ with coping,’ in the sense of minimising the consequences of an adversity and managing vulnerability to ensure short-term survival (Obrist, Pfeiffer, and Henley 2010, 290). Indeed, certain women resume work activities at the expense of restorative rest or despite chronic illness. Their practices exemplify a form of coping that can be construed as a rational response in the short term, but which may actually erode resilience in the long term.

Women's practices and narratives in the aftermath of severe reproductive mishaps’ thus reveal how the realms of reproduction and production are inextricably intertwined, and sometimes jeopardise each other, and at other times reinforce each other or compensate for each other's deficit. Within women's everyday lives, reproduction and production both entail sources of potential empowerment and enhancement, as well as potential threats, to health and well-being, thereby challenging the narrow economic estimations of reproductive morbidity that dominate global health discourse.
